# Mortality following myocardial infarction among HIV-infected persons: the Center for AIDS Research Network Of Integrated Clinical Systems (CNICS)

**DOI:** 10.1186/s12916-019-1385-7

**Published:** 2019-07-31

**Authors:** Matthew J. Feinstein, Robin M. Nance, J. A. Chris Delaney, Susan R. Heckbert, Matthew J. Budoff, Daniel R. Drozd, Greer A. Burkholder, James H. Willig, Michael J. Mugavero, William C. Mathews, Richard D. Moore, Joseph J. Eron, Sonia Napravnik, Peter W. Hunt, Elvin Geng, Priscilla Hsue, Inga Peter, William B. Lober, Kristina Crothers, Carl Grunfeld, Michael S. Saag, Mari M. Kitahata, Donald M. Lloyd-Jones, Heidi M. Crane

**Affiliations:** 1 Division of Cardiology, Department of Medicine, Northwestern University Feinberg School of Medicine, 680 N. Lake Shore Drive, Suite 1400, Chicago, IL 60611 USA; 2 University of Washington School of Medicine, Seattle, USA; 3 School of Public Health, University of Washington, Seattle, USA; 4 University of California-Los Angeles School of Medicine, Los Angeles, USA; 5 University of Alabama-Birmingham School of Medicine, Birmingham, USA; 6 Department of Medicine, University of California-San Diego Medical Center, San Diego, USA; 7 Johns Hopkins University, Baltimore, USA; 8 University of North Carolina School of Medicine, Chapel Hill, USA; 9 University of California-San Francisco School of Medicine, San Francisco, USA; 10 Mount Sinai School of Medicine, New York City, USA

**Keywords:** Human immunodeficiency virus, Myocardial infarction, Mortality, Comorbidity, Cardiovascular diseases, Multicenter study, Epidemiology

## Abstract

**Background:**

Persons with human immunodeficiency virus (HIV) have higher risks for myocardial infarction (MI) than the general population. This is driven in part by higher type 2 MI (T2MI, due to coronary supply-demand mismatch) rates among persons with HIV (PWH). In the general population, T2MI has higher mortality than type 1 MI (T1MI, spontaneous and generally due to plaque rupture and thrombosis). PWH have a greater burden of comorbidities and may therefore have an even greater excess risk for complication and death in the setting of T2MI. However, mortality patterns after T1MI and T2MI in HIV are unknown.

**Methods:**

We analyzed mortality after MI among PWH enrolled in the multicenter, US-based Centers for AIDS Research Network of Integrated Clinical Systems (CNICS) cohort (*N* = 28,186). Incident MIs occurring between January 1, 1996, and December 31, 2014, were centrally adjudicated and classified as T1MI or T2MI. We first compared mortality following T1MI vs. T2MI among PWH. Cox survival analyses and Bayesian model averaging were then used to evaluate pre-MI covariates associated with mortality following T1MI and T2MI.

**Results:**

Among the 596 out of 28,186 PWH who experienced MI (2.1%; 293 T1MI and 303 T2MI), mortality rates were significantly greater after T2MI (22.2/100 person-years; 1-, 3-, and 5-year mortality 39%, 52%, and 62%) than T1MI (8.2/100 person-years; 1-, 3-, and 5-year mortality 15%, 22%, and 30%). Significant mortality predictors after T1MI were higher HIV viral load, renal dysfunction, and older age. Significant predictors of mortality after T2MI were low body-mass index (BMI) and detectable HIV viral load.

**Conclusions:**

Mortality is high following MI for PWH and substantially greater after T2MI than T1MI. Predictors of death after MI differed by type of MI, reinforcing the different clinical scenarios associated with each MI type and the importance of considering MI types separately.

**Electronic supplementary material:**

The online version of this article (10.1186/s12916-019-1385-7) contains supplementary material, which is available to authorized users.

## Background

The global prevalence of human immunodeficiency virus (HIV) and burden of cardiovascular diseases among persons with HIV (PWH) are increasing [1, 2]. Compared with uninfected persons, PWH have greater risks for myocardial infarction (MI) [3–6]. Although several studies have evaluated HIV-related factors and traditional cardiovascular risk factors that contribute to elevated MI risks among PWH [3, 6–10], little is known regarding how these factors may contribute to prognosis after MI in HIV.

Further complicating matters is the difference in MI presentation in HIV versus in the general population. Type 2 MIs (T2MIs), which are secondary to myocardial supply/demand mismatch rather than primary plaque rupture or thrombosis [11], account for as many as half of HIV-associated MIs [9] but a lower [12] (though increasing [13]) proportion of MIs in the general population. Ascertainment, prevention, and treatment of MIs in the general population have traditionally focused on “classic” athero-thrombotic type 1 MIs (T1MIs) in part because the clear majority of MIs in the general population are T1MIs [14–20]. Comparatively sparse data exist regarding prognosis, treatment, and prevention of T2MIs, prompting investigators call for better understand the biological basis and effective management strategies of T2MI [13]. A recent single-center retrospective study in the general population found that 1-year mortality after T1MI was 12.4% vs. 34.9% for T2MI [21]. This difference was driven in large part by non-cardiovascular causes of death following T2MI. Although T2MI may be a particularly important prognostic marker among PWH, who tend to have a greater chronic disease burden, prognosis after T1MI and T2MI has not been studied among PWH.

Given the need to better understand how the predictors, course, and prognosis of MI subtypes differ among PWH, we developed a unique and rigorous MI adjudication protocol in the Centers for AIDS Research Network of Integrated Clinical Systems that distinguishes between MI subtypes [22]. In these data, we found substantial demographic and clinical differences between PWH with incident T1MI vs. T2MI [12]. However, these analyses did not extend to course after MI. Recent observational studies have evaluated myocardial scar burden [23] and mortality [24, 25] following MI in the population with HIV but these studies did not distinguish MI subtype. Therefore, the purpose of this analysis was to compare mortality following T1MI vs. T2MI among PWH as well as differences in clinical and demographic characteristics associated with mortality by MI type. We hypothesized that mortality would be significantly greater following T2MI compared with T1MI among PWH and that factors that predict mortality would differ between MI subtypes. Specifically, we hypothesized that traditional cardiovascular risk factors most closely predict death following T1MI, whereas poor HIV control most closely predicts death following T2MI.

## Methods

### Cohort, eligibility, and covariate assessment

We used the Centers for AIDS Research Network of Integrated Clinical Systems (CNICS) cohort for all analyses. CNICS includes PWH in clinical care at 8 centers for HIV care across the USA. We included data from the 6 CNICS sites (Johns Hopkins University, University of Alabama at Birmingham, University of California-San Diego, University of California-San Francisco, University of North Carolina-Chapel Hill, and University of Washington) that completed adjudication of all detected incident MIs occurring between January 1, 1996, and December 31, 2014. Covariates in the analysis are drawn from the clinical data from all inpatient and outpatient encounters occurring for all HIV-infected persons at CNICS sites. Data in the CNICS repository include demographic data, HIV transmission risk factors, laboratory test results, such as HIV viral load, CD4+ T cell count, blood chemistries, cholesterol levels, and cardiac biomarkers; prescription medications, such as lipid-lowering therapies, antihypertensive medications, and antiretroviral medications; clinical diagnoses such as diabetes, hypertension, and smoking; patient-reported measures such as depression and alcohol use; and vital status including death dates. Each CNICS site obtained institutional review board approval for CNICS, and written informed consent was obtained from all CNICS participants. Demographic and clinical characteristics used for analyses were derived from the most recent visit prior to MI. There was no cap on the duration of time between the most recent visit at which a covariate was measured and MI with the exception of HIV viral load and CD4 T cell count, which were required within 3 years prior to MI for inclusion in this analysis. Otherwise, we carried forward the most recent pre-MI values over whatever time frame was appropriate. The median time between the pre-MI covariate values and MI was 2 months (mean 4 months).

### Myocardial infarction adjudication

Procedures for screening and adjudication of MIs have been described in detail [12, 22]. Possible MIs were identified by a screen for clinical MI diagnoses, coronary intervention documentation, or elevated cardiac biomarker levels. Each CNICS site also requested relevant medical records for any of their participants with reported or suspected MIs at outside hospitals. Sites then assembled de-identified packets of clinical notes, relevant cardiovascular and imaging studies, and laboratory tests which were reviewed independently by two expert physician adjudicators. Antiretroviral medication names were redacted to eliminate the potential for biasing reviewers based on perceived associations between specific antiretroviral medications and MIs. Two expert cardiologist adjudicators independently reviewed these data to determine whether CNICS participants had definite, probable, or no MI, and subsequently categorized events as T1MI or T2MI. Determination of MI presence and subtype included electrocardiographic criteria of evolving Q waves, ST elevations, and new left bundle branch block with specific algorithms used in the presence or absence of chest pain and cardiac biomarker elevations to determine whether MIs were definite, probable, or not present. Reviewers categorized MIs as T1MI or T2MI; definition of T2MI required identification of a clinical cause leading to the T2MI, such as an MI occurring during sepsis. A third expert cardiologist adjudicator was used to resolve discrepancies. Type 3 MIs were not included because these occur without available biomarkers, and there were < 10 MIs in the cohort that would have been categorized as type 4 or 5 MIs; thus, only type 1 and 2 MIs were included. Coronary interventions such as coronary artery bypass graft surgery were combined with T1MI. Only the first MI was included for analysis for CNICS participants with multiple MIs.

### Mortality ascertainment

Deaths were ascertained for all CNICS participants at the local, state, and national level using multiple approaches including state death certificate data and national death indexes.

### Statistical analysis

Follow-up started at the date of the MI and ended at the earliest of the date of death or date of censoring. The date of censoring was 18 months before the latest recorded death at each site in order to adequately ensure capture of deaths, given potential delays in death index data. All covariates were collected at least 7 days before the MI date to preclude MI-related treatment or laboratory changes to covariates. Due to this covariate collection date requirement, only MIs at least 7 days after the initial CNICS visit date were included. Regarding missing data, there were a total of 11 participants excluded because they did not have complete HIV viral load and CD4 T cell count data within 3 years prior to MI; the only other variable with missing data was body-mass index (BMI), which was 5.7% missing and for which missing data were handled with multiple imputation.

We used 3 approaches to identify potential predictors of death. First, Cox survival models were used to investigate the association of MI with covariates that were chosen a priori as clinically important. Second, to allow for variable selection into a more parsimonious statistical model and decide between potentially collinear predictors, Bayesian model averaging [26, 27] as applied to Cox models [28] was used to select potentially important covariates. We used a threshold of > 30% posterior probability of a predictor being included in the statistical model based on the goodness of fit of the family of possible models. Once the predictors were selected using Bayesian model averaging, Cox survival models with the selected covariates with high posterior probabilities of selection were used to estimate the associated hazard ratios for T1MI and T2MI. This was our primary analysis, as it allows a principled approach to selecting among highly correlated covariates. We also performed sensitivity analyses in which we repeated these analyses excluding persons who died within 30 days after MI in order to better isolate MI as an exposure rather than a near-concurrent outcome with death. Given the possibility that sepsis would precipitate many T2MIs and would affect post-MI mortality, we also performed a sensitivity analysis of mortality following T2MIs in which persons with and without sepsis were analyzed separately.

## Results

Of 28,186 PWH from across the USA eligible for analyses, 596 (2.1%) experienced MIs (293 T1MIs and 303 T2MIs: Table 1). Compared to people with T1MI, people with T2MI were more likely to be female and African-American; they were also more likely to use injection drugs, less likely to take antiretroviral therapy, and had lower CD4 counts and higher HIV viral loads prior to MI than participants with T1MI (data not shown as we have published these previously [12]). The most common cause of T2MI was sepsis or bacteremia, which accounted for 35% of T2MI; the next most common causes were cocaine or other illicit drug use (14% of T2MI), followed by hypertensive urgency or emergency (10%) and respiratory failure (9%) [12].

**Table 1:** Demographics and clinical characteristics for persons with HIV by mortality status following myocardial infarction

	MI		
Variable: *N* (%) or mean (SD)*	Type 1	Type 2	*P* value
*N*	293	303	
Age	51 (9)	49 (11)	0.02
Female (%)	49 (17)	82 (27)	0.002
Ethnicity			< 0.001
White	149 (51)	73 (24)	
Black	115 (39)	205 (68)	
Hispanic	20 (7)	18 (6)	
Other/missing	9 (3)	7 (2)	
Log_10_(HIV viral load (VL) + 1)	2.4 (1.4)	3.1 (1.6)	< 0.001
Log_10_(max VL + 1)	4.8 (1.1)	4.9 (1.1)	0.3
CD4	436 (304)	333 (292)	< 0.001
Nadir CD4	174 (174)	168 (201)	0.7
BMI**			< 0.001
< 18.5	13 (4)	31 (10)	
18.5 to < 25	111 (38)	138 (46)	
25 to < 30	100 (34)	64 (21)	
≥ 30	60 (20)	45 (15)	
Missing BMI	9 (3)	25 (8)	
Diabetes	74 (25)	69 (23)	0.5
eGFR < 30 mL/min/1.73m^2^	33 (11)	51 (17)	0.05
Statin use	84 (29)	41 (14)	< 0.001
Treated hypertension	98 (33)	93 (31)	0.5
Smoker	134 (46)	130 (43)	0.5

*Demographic and clinical characteristics are derived from the most recent visit prior to MI

**9 people with type 1 MI were missing BMI measurements (7 of whom died during follow-up) and 25 people with type 2 MI were missing BMI measurements (19 of whom died during follow-up)

**Fig. 1:**
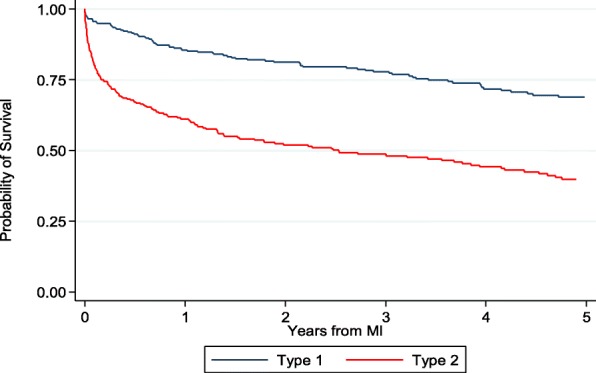
Survival following type 1 and type 2 myocardial infarction among HIV-infected patients

Mortality rates were significantly greater following T2MI (22.2 deaths per 100 person-years; 54.1% overall) than T1MI (8.2 deaths per 100 person-years; 36.2% overall) (Table 2). Participants with T2MI were particularly likely to die early during follow-up; mortality at 1, 3, and 5 years after MI was 39%, 52%, and 62% for T2MI versus 15%, 22%, and 30% for T1MI (Fig. 1).

**Table 2:** Demographics and clinical characteristics for persons with HIV by mortality status following myocardial infarction

	Type 1 MI			Type 2 MI		
Variable: *N* (%) or mean (SD)*	Lived	Died	*P* value	Lived	Died	*P* value
*N*	187	106		139	164	
Age	50 (9)	52 (9)	0.05	50 (11)	48 (11)	0.1
Female (%)	32 (17)	17 (16)	0.8	35 (25)	47 (29)	0.5
Ethnicity			0.2			0.2
White	102 (55)	47 (44)		35 (25)	38 (23)	
Black	65 (35)	50 (47)		90 (65)	115 (70)	
Hispanic	15 (8)	5 (5)		12 (9)	6 (4)	
Other/missing	5 (3)	4 (4)		2 (1)	5 (3)	
Log_10_(HIV viral load (VL) + 1)	2.2 (1.4)	2.8 (1.5)	0.001	2.8 (1.6)	3.3 (1.6)	0.002
Log_10_(max VL + 1)	4.8 (1.1)	4.8 (1.2)	0.9	4.8 (1.1)	5.0 (1.1)	0.04
CD4	472 (313)	372 (276)	0.005	361 (291)	310 (291)	0.1
Nadir CD4	186 (184)	154 (154)	0.1	185 (224)	153 (179)	0.2
BMI**			0.005			0.002
< 18.5	5 (3)	8 (8)		8 (6)	23 (14)	
18.5 to < 25	71 (38)	40 (38)		66 (47)	72 (44)	
25 to < 30	63 (34)	37 (35)		39 (28)	25 (15)	
≥ 30	46 (25)	14 (13)		20 (14)	25 (15)	
Missing BMI	2 (1)	7 (7)		6 (4)	19 (12)	
Diabetes	44 (24)	30 (28)	0.4	31 (22)	38 (23)	0.9
eGFR < 30 mL/min/1.73m^2^	15 (8)	18 (17)	0.02	15 (11)	36 (22)	0.01
Statin use	52 (28)	32 (30)	0.7	24 (17)	17 (10)	0.08
Treated hypertension	63 (34)	35 (33)	0.9	52 (37)	41 (25)	0.02
Smoker	93 (50)	41 (39)	0.07	61 (44)	69 (42)	0.8

*Demographic and clinical characteristics are derived from the most recent visit prior to MI

**9 people with type 1 MI were missing BMI measurements (7 of whom died during follow-up) and 25 people with type 2 MI were missing BMI measurements (19 of whom died during follow-up)

Among participants with T1MI, those who died during follow-up were significantly older and had significantly higher HIV viral loads, lower CD4 counts, and lower body-mass index (BMI) prior to MI than participants who did not die during follow-up (Table 2). Among participants with T2MI, those who died during follow-up had significantly higher HIV viral load, lower BMI, and lower estimated glomerular filtration rate (eGFR) prior to their MI compared with those who did not die during follow-up. On univariate analyses used for model selection, older age was associated with a significantly elevated risk for death after T1MI but not after T2MI (Table 3). Black race was associated with a borderline higher risk for death after T1MI but not T2MI in univariate analyses, but was no longer a significant predictor in multivariable analyses or when Bayesian model averaging was used to select significant predictors of post-MI mortality. Factors significantly associated with elevated rates of death following T1MI were eGFR < 30 mL/min/1.73m^2^, older age, and higher viral load prior to MI (Table 3). Meanwhile, for T2MI, only BMI < 18.5 kg/m^2^ was associated with a significantly elevated hazard of death and BMI 25–30 kg/m^2^ was associated with a significantly lower hazard of death (Table 3).

**Table 3:** Hazard ratios for mortality among persons with HIV following type 1 and type 2 myocardial infarction: univariate regression

Variable	Hazard ratio for death (95% confidence interval)	*P* value	Hazard ratio for death (95% confidence interval)	*P* value
	Type 1 MI		Type 2 MI	
Antiretroviral use	0.62 (0.39–0.98)	0.04	0.74 (0.54–1.01)	0.06
Female sex	1.03 (0.61–1.73)	0.92	1.23 (0.88–1.73)	0.23
Age (per 10 years older)	1.54 (1.23–1.93)	< 0.01	0.91 (0.79–1.05)	0.20
Ethnicity				
Black	1.49 (1.01–2.18)	0.04	1.07 (0.76–1.49)	0.71
Hispanic	0.84 (0.34–2.08)	0.71	0.55 (0.24–1.24)	0.15
Other/missing	1.51 (0.55–4.11)	0.42	1.68 (0.69–4.10)	0.25
CD4 (per 100)	0.93 (0.86–1.00)	0.05	0.96 (0.90–1.01)	0.12
Log10(VL + 1)	1.16 (1.02–1.32)	0.02	1.11 (1.01–1.22)	0.03
Diabetes	1.49 (0.97–2.28)	0.07	1.03 (0.72–1.48)	0.87
eGFR < 30 mL/min/1.73m^2^	2.51 (1.50–4.22)	< 0.01	1.30 (0.90–1.89)	0.16
Treated hypertension	1.12 (0.74–1.68)	0.59	0.66 (0.46–0.94)	0.02
Smoker	0.76 (0.51–1.12)	0.17	0.97 (0.71–1.32)	0.83
BMI < 18.5 kg/m^2^	1.66 (0.82–3.35)	0.16	1.86 (1.21–2.88)	< 0.01
BMI 25 to < 30 kg/m^2^	0.98 (0.65–1.46)	0.91	0.50 (0.33–0.77)	< 0.01
BMI ≥ 30 kg/m^2^	0.61 (0.36–1.04)	0.07	1.11 (0.73–1.69)	0.61

When we used the Bayesian Model Averaging to determine factors most likely to predict mortality following MI, several HIV-related factors were associated with post-MI mortality. The predictors of elevated mortality after T1MI were higher pre-MI log_10_ of HIV viral load, eGFR < 30 mL/min/1.73m^2^, and older age (Table 4), all of which had a posterior probability of at least 95% of being in the best fitting statistical model. The predictors of greater mortality after T2MI were body mass index < 18.5 kg/m^2^ and HIV viral load > 400 copies/mL, with posterior probabilities between 30 and 40%; body-mass index 25.0 to 29.9 kg/m^2^ prior to T2MI was associated with a significantly lower hazard of subsequent mortality (Table 4) with a posterior probability greater than 95%.

**Table 4:** Significant predictors of mortality following type 1 and type 2 myocardial infarction among persons with HIV: Bayesian Model Averaging

Variable	Probability of inclusion in the final model (%)	HR*	95% CI*	*P* value	Probability of inclusion in the final model (%)	HR*	95% CI*	*P* value
	Type 1 MI							
Statin use	3.3				4.5			
ART use	8.8				11.5			
Female	2.9	0.91	0.53, 1.55	0.723	6.7	1.24	0.88, 1.75	0.219
Diabetes	13.9				3.4			
Treated hypertension	2.0				19.9			
Age (per 10)	*100.0*	*1.73*	*1.37, 2.17*	*< 0.001*	2.5	0.97	0.84, 1.12	0.664
Black	10.8				1.8			
Hispanic	3.2				6.3			
Smoker	7.6				1.8			
Log10(VL + 1)	*95.1*	*1.25*	*1.09, 1.43*	*0.001*	21.0			
CD4 (per 100)	4.5				4.3			
VL > 400	24.4				*33.6*	*1.38*	*1.00, 1.91*	*0.047*
eGFR < 30	*100.0*	*3.02*	*1.77, 5.14*	*< 0.001*	11.1			
BMI < 18.5	9.1				*37.9*	*1.62*	*1.02, 2.56*	*0.040*
BMI 25 to < 30	1.9				*96.7*	*0.55*	*0.35, 0.86*	*0.009*
BMI 30+	20.0				1.8	1.05	0.68, 1.64	0.819

*Hazard ratio and confidence interval from Cox model included for potential significant predictors plus age and sex

**Fig. 2:**
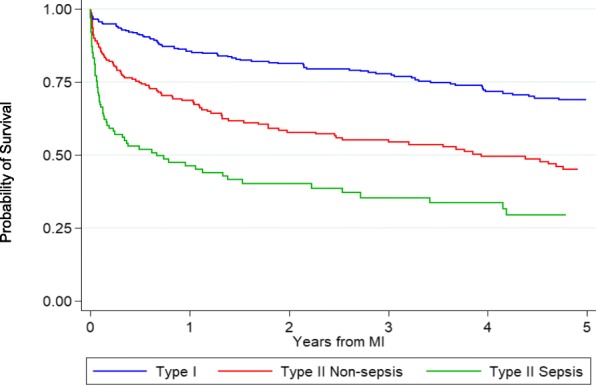
Survival following type 1 myocardial infarction, type 2 myocardial infarction due to sepsis, and type 2 myocardial infarction not due to sepsis among HIV-infected patients

In sensitivity analyses excluding participants who died within 30 days of MI, eGFR < 30 mL/min/1.73m^2^ and older age were significant predictors of mortality following T1MI (Additional file 1: Table S1). After T2MI, eGFR < 30 mL/min/1.73m^2^ and BMI < 18.5 kg/m^2^ were associated with significantly elevated mortality (Additional file 1: Table S1). When we analyzed mortality after T2MI separately for persons with and without sepsis, the mortality rates were greater among those with sepsis, but mortality for persons with T2MI without sepsis was still substantially greater than mortality for persons with T1MI (Fig. 2).

## Discussion

We evaluated predictors of mortality following MI in a large, multicenter HIV cohort with long-term follow-up and rigorous MI adjudication. We found that mortality is high for PWH after MI and, similar to the general population, mortality is particularly high after T2MI. We found that one-year mortality for PWH was 15% after T1MI and 39% after T2MI—these were similar to a recent report from the general population (12.4% and 34.9% 1-year mortality after T1MI and T2MI, respectively) [21]. Nevertheless, our observed mortality rates after T1MI for PWH were generally higher than those observed after acute coronary syndromes or percutaneous coronary intervention for PWH in other, primarily European, cohorts [25, 29–31]. While this is somewhat expected because we analyzed mortality after MI only and did not include people with non-MI acute coronary syndromes (which are by definition less severe and associated with less morbidity and mortality), differences in clinical and behavioral risk factor burden, as well as clinical management and access to care, between PWH in the United States and Europe may also underlie the differences in observed post-MI mortality rates. We found that older age was associated with significantly greater mortality risk following T1MI but not T2MI, whereas for T2MI, low BMI was associated with a significantly greater mortality risk. Renal dysfunction prior to MI was associated with significantly elevated mortality regardless of MI type.

Perhaps the most likely explanation for the finding that older age was associated with mortality after T1MI but not T2MI relates to the different underlying demographic and clinical factors associated with T1MI versus T2MI. T1MIs generally result from atheromatous coronary plaque development and overlying thrombosis; this plaque burden reflects decades’ worth of exposure to cardiovascular disease (CVD) risk factors (such as diabetes, hypertension, smoking, and dyslipidemia, among others) that contribute fairly predictably to vascular aging. Older PWH with T1MI may thus tend to be globally sicker than younger HIV-infected persons with T1MI given their accumulated longitudinal exposure to CVD risk factors and comorbidities, which are themselves associated predictably with morbidity and mortality. It may also suggest that younger age was less protective against mortality in the T2MI population, given that the mortality rate was higher among the T2MIs than the T1MIs (Fig. 1). Even more striking, the mean age was lower for fatal T2MIs than non-fatal T2MIs (Table 1), suggesting that a diagnosis of T2MI is an indicator of more severe underlying comorbidity associated with mortality at younger ages.

Although patients with T2MI may have high cumulative exposure to CVD risk factors, they are substantially more likely to have severe non-cardiovascular comorbidities that are not as clearly or predictably associated with aging [19]. Thus, PWH with T2MI may be similarly “sick” regardless of age, as their extent of comorbidity may not be due to long-term accumulation of aging-associated exposures. The steep mortality slope apparent for participants with T2MI due to sepsis (compared with T1MI and, to a lesser extent, T2MI not due to sepsis) suggests that a substantial portion of the post-MI mortality among those with T2MI, particularly T2MI due to sepsis, relates to the event (e.g., sepsis) associated with the T2MI.

The differences we observed in predictors of mortality following T1MI vs. T2MI reflect the diverse pathophysiologies and concomitant comorbidities associated with these different MI subtypes. The substantially greater mortality after T2MI versus after T1MI for PWH raises the question of whether the difference is driven by (1) critical illnesses (e.g., sepsis) leading to the T2MIs, (2) underlying patient risk factors (e.g., intravenous drug use and poor HIV control) that led to these critical illnesses, and/or (3) cardiac damage incurred during the T2MIs. Unfortunately, T2MI is a heterogeneous condition for which optimal prevention and treatment are far less clear than T1MIs, for which revascularization and pharmacologic strategies have dramatically altered the post-event natural history. This heterogeneity in T2MI triggers may be particularly notable among PWH given the severity of HIV-associated critical systemic illnesses, such as opportunistic infections and associated sepsis. The greater mortality rates following T2MI that we observed among HIV-infected persons with low BMI (compared with normal BMI) are not surprising given the association of low BMI with mortality in general, and likely reflect the known greater burden of HIV-associated wasting, malnourishment, and/or concomitant high-risk behaviors (e.g., intravenous drug use) among underweight PWH [32–34].

Our findings should be interpreted in the context of this study’s limitations. A key premise of this study is that T1MI and T2MI are fundamentally different; there is heterogeneity in the pathophysiology of T2MIs as well as how they are defined and assessed [14, 35]. To ensure optimal validity in MI ascertainment, MIs were adjudicated by two independent cardiologists (and a third where disagreements occurred) using rigorous and pre-specified criteria. Another limitation of this study is the possibility that MIs occurring outside of CNICS sites were not ascertained. We sought to address this potential limitation by asking sites to request relevant medical records for any of their participants with reported or suspected MIs at outside hospitals. As with any observational study, we were unable to infer causality between exposures at the time of MI and subsequent mortality risk. Additionally, because valid cause-specific mortality data are not available in CNICS at this time, we were unable to assess underlying causes of death in this analysis. We were also unable to comprehensively define differences in mortality between CNICS sites; such differences are certainly possible given differences in demographics and CVD risk factors across CNICS sites. Nevertheless, the CNICS cohort represents a fundamental strength of this analysis, as it is a large, geographically and ethnically diverse cohort that has prospectively collected comprehensive clinical data on HIV-infected persons receiving clinical care for nearly two decades. This underscores the generalizability of our findings to PWH in clinical care more generally. Finally, our approaches to variable selection allowed us to decide between potentially quite collinear covariates to build the model most consistent with the underlying data.

## Conclusions

We found that post-MI mortality rates for PWH are elevated in general and particularly elevated after T2MI, in a diverse, multi-center cohort of over 28,000 PWH with rigorous MI adjudication. The relative difference we found in mortality after T1MI and T2MI for PWH was similar to that of uninfected persons. High HIV viral load was associated with death after T1MI and T2MI, underscoring the importance of HIV viral suppression even in the setting of competing risks for non-AIDS events such as MI. We also found that older age is associated with greater mortality following T1MI, but not T2MI, whereas low BMI is associated with greater mortality following T2MI. These differences reflect the clinical, demographic, and pathophysiological differences between T1MI and T2MI and may be particularly relevant given the high proportion of MIs in HIV that are type 2. Further studies are needed to identify optimal approaches to preventing morbidity and mortality after MI among PWH.

## Additional file

Additional file 1: **Table S1.** Significant predictors of mortality > 30 days following myocardial infarction among persons with HIV: Cox models with Bayesian model averaging to select variables. (DOCX 19 kb)

## Data Availability

The datasets generated and/or analyzed during the current study are not publicly available due to institutional data use guidelines, but are available from the authors on reasonable request.

## Abbreviations

BMI: Body mass index
CNICS: Centers for AIDS Research Network of Integrated Clinical Systems
CVD: Cardiovascular disease
eGFR: Estimated glomerular filtration rate
HIV: Human immunodeficiency virus
MI: Myocardial infarction
PWH: Persons with human immunodeficiency virus
T1MI: Type 1 myocardial infarction
T2MI: Type 2 myocardial infarction
