# Supplementary material for: Mortality following myocardial infarction among HIV-infected persons: the Center for AIDS Research Network Of Integrated Clinical Systems (CNICS)
Source: BMC Med. 2019 Jul 31;17:149. doi: 10.1186/s12916-019-1385-7 (PMC6668167; doi:10.1186/s12916-019-1385-7)
Supplement: Supplementary file 1 — Table S1. Significant predictors of mortality > 30 days following myocardial infarction among persons with HIV: Cox models with Bayesian model averaging to select variables. (DOCX 19 kb) [file 12916_2019_1385_MOESM1_ESM.docx]

Additional file 1: Table S1

| **Variable** | **Probability of inclusion in final model (%)** | **HR*** | **95% CI*** | **P-value** |
| --- | --- | --- | --- | --- |
| **Type 1 MI** | | | | |
| Statin use | 3.0 |  |  |  |
| ART use | 9.8 |  |  |  |
| Female | 3.0 | 0.94 | 0.54, 1.65 | 0.834 |
| Diabetes | 24.7 |  |  |  |
| Treated Hypertension | 2.2 |  |  |  |
| **Age (per 10)** | **100.0** | **1.70** | **1.33, 2.18** | **<0.001** |
| Black | 14.5 |  |  |  |
| Hispanic | 3.1 |  |  |  |
| Smoker | 11.3 |  |  |  |
| **Log10(VL+1)** | **90.1** | **1.24** | **1.07, 1.43** | **0.004** |
| CD4 (per 100) | 4.7 |  |  |  |
| VL >400 | 28.4 |  |  |  |
| **eGFR<30** | **100.0** | **3.88** | **2.23, 6.73** | **<0.001** |
| BMI <18.5 | 12.1 |  |  |  |
| BMI 25-<30 | 3.4 |  |  |  |
| BMI 30+ | 9.4 |  |  |  |
| **Type 2 MI** | | | | |
| Statin use | 3.0 |  |  |  |
| ART use | 29.1 |  |  |  |
| Female | 12.5 | 1.42 | 0.93, 2.16 | 0.105 |
| Diabetes | 2.6 |  |  |  |
| Treated Hypertension | 2.7 |  |  |  |
| Age (per 10) | 4.0 | 0.94 | 0.78, 1.13 | 0.528 |
| Black | 2.6 |  |  |  |
| Hispanic | 7.8 |  |  |  |
| Smoker | 5.7 |  |  |  |
| Log10(VL+1) | 4.5 |  |  |  |
| CD4 (per 100) | 3.7 |  |  |  |
| VL >400 | 6.3 |  |  |  |
| **eGFR<30** | **96.6** | **2.02** | **1.33, 3.08** | **0.001** |
| **BMI <18.5** | **100.0** | **2.71** | **1.60, 4.58** | **<0.001** |
| BMI 25-<30 | 7.5 | 0.79 | 0.48, 1.32 | 0.371 |
| BMI ≥30 | 13.2 | 1.38 | 0.80, 2.38 | 0.246 |
